# Longitudinal cognitive outcomes in two progressive supranuclear palsy clinical trials

**DOI:** 10.1002/alz.71641

**Published:** 2026-06-26

**Authors:** Zoë C. Cooper, Indira Garcia‐Cordero, Maria Carmela Tartaglia, Adam M. Staffaroni, Kevin Duff, Douglas Gunzler, Adam L. Boxer, Anne‐Marie Wills

**Affiliations:** ^1^ Department of Neurology Massachusetts General Hospital Boston Massachusetts USA; ^2^ Cognitive Neuroscience Center Universidad de San Andrés Buenos Aires Argentina; ^3^ National Scientific and Technical Research Council (CONICET) Buenos Aires Argentina; ^4^ Tanz Centre for Research in Neurodegenerative Diseases University of Toronto Toronto Ontario Canada; ^5^ Krembil Brain Institute University Health Network Toronto Ontario Canada; ^6^ Fein Memory and Aging Center Department of Neurology Weill Institute for Neurosciences University of California San Francisco San Francisco California USA; ^7^ Layton Aging & Alzheimer's Disease Center Oregon Health & Science University Portland Oregon USA; ^8^ Population Health and Equity Research Institute Center for Health Care Research and Policy MetroHealth Medical Center School of Medicine Case Western Reserve University Cleveland Ohio USA; ^9^ Department of Neurology Harvard Medical School Massachusetts General Hospital Boston Massachusetts USA

**Keywords:** cognition, cognitive decline, Color Trails, executive function, fluency, memory, progressive supranuclear palsy (PSP), Repeatable Battery for the Assessment of Neuropsychological Status (RBANS), visuospatial function

## Abstract

**INTRODUCTION:**

Progressive supranuclear palsy (PSP) causes executive dysfunction, fluency deficits, and behavioral changes. We examined longitudinal changes in PSP cognition using the Repeatable Battery for the Assessment of Neuropsychological Status (RBANS).

**METHODS:**

Using the RBANS and executive function and fluency tests from 486 and 377 participants in the PASSPORT (NCT03068468) and tilavonemab (NCT02985879) clinical trials, we assessed linear mixed models of cognitive subtest score change, controlling for disease duration, age, sex, and treatment group.

**RESULTS:**

The greatest declines occurred in subtests assessing visuospatial (figure copy, figure recall), executive function (Color Trails, coding), and fluency (primarily semantic fluency), although controlling for motor and ocular motor disability reduced the magnitude of score decline. Subtests assessing immediate and delayed memory (story recall, story memory, list recall, list recognition) declined slowly or not at all.

**DISCUSSION:**

Cognition in PSP is characterized by declines in executive, visuospatial function, and fluency, with relative preservation of memory.

## Highlights

- Executive and visuospatial function and fluency (semantic) decline fastest in PSP.
- Memory subtest scores show minimal, often non‐significant, change over time.
- Motor and oculomotor disability contribute to declining scores on cognitive tests.

## 1 BACKGROUND

Progressive supranuclear palsy (PSP) is a rare and rapidly progressive neurological condition characterized by postural instability with falls, generalized bradykinesia, impaired ocular movements, and dysphagia.^1^, ^2^, ^3^, ^4^ Cognitive deficits include dysexecutive symptoms (difficulty planning and organizing), bradyphrenia, and impaired phonemic (letter) fluency, and PSP can be associated with behavioral changes such as apathy and impulsivity.^5^, ^6^, ^7^, ^8^ Conversely, orientation and memory are less impaired compared to Alzheimer's disease.^6^

Characterizing cognitive deficits and longitudinal cognitive decline in PSP is essential for understanding disease progression and tracking changes during clinical trials. Previous studies used a range of outcomes to measure cognitive dysfunction, although many had limitations. For example, global measures such as the Revised Addenbrooke's Cognitive Examination reliably differentiate PSP from Parkinson's disease (PD)^9^ and Alzheimer's disease.^10^ Its verbal fluency and visuospatial components exhibit decline, but the total score does not change appreciably.^9^, ^11^ The Mini‐Mental State Examination (MMSE) also shows significant 1‐year decline^12^ but is limited by ceiling effects.^9^, ^13^ Both measures emphasize memory and orientation and are less sensitive to executive dysfunction. The Montreal Cognitive Assessment shows significant but small declines in attention, language, naming, orientation, and visuospatial/executive skills^14^ and reliably differentiates between PSP and PD.^13^ Executive function has been assessed using the Frontal Assessment Battery (FAB)^15^ and the Color Trails Tests, measures of visual attention and executive processing.^16^ The FAB captures PSP cognitive deficits but does not reliably measure cognitive change.^6^, ^11^ Color Trails correlate strongly with midbrain atrophy and motor clinical scales but are subject to a floor effect.^17^, ^18^ As PSP clinical trials expand, there is a growing need for a brief, repeatable battery of cognitive tests that is sensitive to cognitive change.

Recently, the Repeatable Battery for Assessment of Neuropsychological Status (RBANS) has been incorporated into large PSP clinical trials. The RBANS comprises 12 subtests across five domains: immediate memory, delayed memory, visuospatial/construction, language, and attention.^19^ In the davunetide trial of 313 patients with PSP,^20^ significant baseline cognitive deficits were observed, with mean Total Scale scores falling at the fourth percentile compared to cognitively intact older adults.^4^, ^18^, ^20^ Impairments were most pronounced in visuospatial/construction, attention, and language indices.^4^ RBANS Total Scale score correlated modestly with measures of PSP physical and functional disability, including the PSP rating scale (PSPRS)^21^ and Schwab and England Activities of Daily Living scale,^4^ with lower cognitive scores predicting poorer ratings on the PSPRS.^22^ Prior research showed measurable RBANS decline in PSP over 6‐ and 12‐month periods. Over 1 year, the Total Scale score declined by an average of six standard score points (i.e., 4/10 of a standard deviation), albeit with substantial inter‐patient variability.^18^, ^20^ Examination of the RBANS indices revealed that the language index declined most rapidly over 6 months, while visuospatial/construction and delayed memory declined least.^23^ Between months 6 and 12, the visuospatial/construction and attention indices exhibited the largest additional declines. Compared to regression‐based change formulas for cognitively intact older adults,^24^ PSP patients showed greater cognitive decline across all RBANS indices. This difference was most pronounced for the attention, language, and visuospatial/construction indices, with smaller differences for immediate and delayed memory.^23^ The authors lacked access to the individual RBANS subtest scores and acknowledged that subtests may exhibit distinct patterns from indices, as indices were developed a priori and may not best capture PSP cognition.^25^

In this study, we aimed to quantify cognitive decline in PSP using data from the 486 participants in the PASSPORT^26^ and 377 participants in the tilavonemab (ABBV‐8E12)^27^ clinical trials. Both trials included the RBANS, a phonemic fluency test, and Color Trails Tests; the PASSPORT trial also included the Wechsler Letter‐Number sequencing test. We studied the 16 individual cognitive tests (12 RBANS subtests and four additional cognitive tests) and their rates of decline across two large well‐characterized PSP clinical trial cohorts.

## 2 METHODS

### 2.1 Study participants and design

Cognitive and motor disability data were obtained from two large PSP clinical trials: the Study of BIIB092 in Participants with PSP (PASSPORT; ClinicalTrials.gov identifier: NCT03068468) ^26^ and the Study of ABBV‐8E12 in Subjects with PSP (ClinicalTrials.gov identifier: NCT02985879).^27^ Both were phase 2, randomized, double‐blind, placebo‐controlled trials.

The PASSPORT trial evaluated the safety and efficacy of gosuranemab, a monoclonal antibody targeting N‐terminal tau.^26^ A total of 486 participants with PSP were enrolled across 90 outpatient sites and 13 countries. The primary endpoint – the adjusted mean change of PSPRS score from baseline to week 52 between gosuranemab and placebo – was not met; therefore, treatment arms were pooled for this secondary analysis. This study includes all participants with at least one baseline cognitive test score (*n* = 486) and includes data from baseline through week 52. Eligibility criteria included probable or possible PSP as defined by National Institute of Neurological Disorders and Stroke and Society for PSP (NINDS‐SPSP) criteria,^28^ symptom onset ≤ 5 years before screening, a score of ≥ 20 on the MMSE, and living outside of a skilled nursing or dementia care facility. Clinical trial data were obtained through the Rare Disease Cures Accelerator—Data and Analytics Platform (RDCA‐DAP) and administered by the Critical Path Institute with permission from Biogen.

The Study of ABBV‐8E12 tested the safety and efficacy of tilavonemab, an N‐terminal tau‐targeting monoclonal antibody.^27^ The trial terminated early after meeting prespecified interim futility criteria, so treatment arms were pooled in the present analysis (*n* = 377 at baseline; 151 participants completed week 52). Eligibility criteria were comparable between the two trials. Enrolled participants met the NINDS‐SPSP diagnosis criteria for probable or possible PSP,^28^ had symptom onset ≤ 5 years before screening, scored ≥ 15 on the MMSE, and lived outside a skilled nursing or dementia care facility. This study was based on research using data from data contributor AbbVie that have been made available through Vivli Inc.^29^ Data are available (https://vivli.org/) with the permission of Vivli Inc.

### 2.2 Cognitive outcome measures

In the PASSPORT trial, the RBANS was administered at weeks 0, 12, 24, 36, and 52. The four additional cognitive function tests – Color Trails 1 and 2, Wechsler Letter‐Number Sequencing, and phonemic (letter) fluency – were administered at weeks 0, 12, 24, 36, and 48. To mitigate practice effects, four versions of the RBANS and Color Trails (A, B, C, D) were administered sequentially, such that version A was repeated at week 52 (RBANS) or 48 (Color Trails). Three versions of the phonemic fluency test (letters F–L; T–S; and R–M) were administered sequentially, with the F–L and T–S versions repeated at weeks 36 and 48, respectively. Measurement of participant limb motor and ocular motor disability was derived from the limb motor and ocular motor subsections of the PSPRS.

The tilavonemab trial included the RBANS, Color Trails 1 and 2, and phonemic fluency, but not Wechsler Letter‐Number Sequencing. Cognitive tests were completed at weeks 0, 8, 20, 32, and 48. Limb motor and ocular motor disability scores come from the PSPRS.

The PASSPORT trial dataset served as the primary dataset for this analysis due to its larger sample size and duration and its inclusion of more cognitive tests. The tilavonemab trial dataset was used to verify key results.

**RESEARCH IN CONTEXT**

1. **Systematic review**: We reviewed existing literature on cognitive scales in PSP using PubMed searches of keywords: “PSP,” “cognition,” “longitudinal,” “executive,” “fluency,” “visuospatial,” and “RBANS.” While prior studies have analyzed cognition, often in small cohorts, few have examined longitudinal change. Furthermore, studies have examined decline in RBANS Total Scale score and indices but not within individual subtests, which may better measure cognition in PSP.
2. **Interpretation**: Our study is the largest prospective study of well‐defined PSP cohorts using a comprehensive set of assessments of cognitive domains. Our findings indicate that PSP patients experience significant declines in executive and visuospatial function and semantic fluency. Motor and ocular motor impairment hinder assessment of cognitive decline.
3. **Future directions**: Future work should assess cognitive decline across both earlier and later stage PSP. Visuospatial outcome measures less sensitive to motor and ocular motor impairment are needed.

### 2.3 Statistical analyses

We examined longitudinal score change across 16 cognitive subtests (12 RBANS subtests and four additional cognitive function tests) using linear mixed‐effects models (LMMs). Scores from Color Trails 1 and 2 were recoded so that lower values represent worse cognitive ability. Scores were standardized as *z*‐scores within each cognitive test using the pooled study sample across all participants and visits – rather than external normative data – to enable between‐measure comparisons. Each fitted LMM incorporated fixed effects for participant sex, baseline age group (categorized in 5‐year intervals since exact participant age was not available), years since diagnosis at baseline, treatment arm, time (in months, modeled as a continuous variable), and participant‐specific random intercepts to account for within‐participant correlation across repeated measurements. Restricted maximum likelihood (REML) was used as the estimation method. REML is robust to mild violations of normality as estimates of variance components tend to be less biased under REML than under full maximum likelihood. Maximum likelihood estimation also handles missing data under the missing at random (i.e., missing dependent on data at hand) assumption, allowing all available cases to be included in the analyses. Bonferroni‐adjusted *p* values were reported for longitudinal cognitive outcome change for a two‐sided alpha of 0.05. The same analysis was completed in both datasets aside from Wechsler Letter‐Number Sequencing, which was not included in the tilavonemab clinical trial. As a sensitivity analysis, LMMs were re‐fitted in the PASSPORT dataset including only participants who completed the double‐blind period of the trial (baseline through week 52). This sensitivity analysis was not completed in the tilavonemab dataset due to early trial termination.

We also compared cognitive scores at baseline and final follow‐up (week 48 or 52) in the PASSPORT dataset using paired *t*‐tests, restricted to participants with cognitive test scores at both timepoints. Effect sizes were quantified using Cohen's *d* (mean difference divided by pooled standard deviation). Histograms were used to visually compare raw scores at each time point. A two‐sided alpha of 0.05 was used throughout, with Bonferroni correction to adjust for multiple comparisons across the 16 outcomes. Wilcoxon signed‐rank tests were conducted as a sensitivity analysis to account for potential non‐normality.

All analyses were completed using RStudio statistical software version 4.4.1. The statistical models were fitted using the lme4 and lmerTest packages.

### 2.4 Sensitivity analysis

Two additional models were fitted to assess cognitive subtest score changes when controlling for motor disability. Primary LMMs were re‐fitted with participant PSPRS limb motor and ocular motor scores included as covariates, separately. We report monthly *z*‐score changes and Bonferroni‐adjusted *p* values. As an exploratory analysis, primary LMMs in the PASSPORT dataset were adjusted for individual PSPRS item‐level scores within limb motor and ocular motor domains. To identify specific PSPRS items that may influence cognitive testing, effect sizes and percent change relative to main model results are reported.

## 3 RESULTS

A total of 486 participants from the PASSPORT trial with at least one cognitive test result were included in the primary analysis. The mean (SD) years since diagnosis was 1.66 (1.34), and the mean baseline age was 68.7 (6.86) years; 210 participants (43.2%) were female (Table 1). Demographic characteristics for the two clinical trial cohorts are comparable (Table 1).^26^, ^27^ In the PASSPORT trial, the cognitive tests with the highest rates of participant non‐completion were Color Trails 1 and 2 (Table 2). At baseline, 371 (76.3%) and 267 (54.9%) participants completed Color Trails 1 and 2, respectively, declining to 248 (58.2%) and 180 (42.3%) by week 48. The only other cognitive test with completion below 90% was the RBANS coding subtest (a digit‐symbol substitution test), for which completion declined from 464 (95.5%) at baseline to 353 (83.6%) at week 52 (Table 2).

**TABLE 1:** Baseline demographics of the PASSPORT and tilavonemab (ABBV‐8E12) clinical trial cohorts.

	PASSPORT (*n* = 486)	Tilavonemab (ABBV‐8E12) (*n* = 377)
Age, years	68.7 ± 6.9	68.8 ± 6.8
Sex, female, *n* (%)	210 (43.2)	158 (41.9)
Race, white, *n* (%)	419 (86.2)	324 (85.9)
Time since symptom onset, years	3.24 ± 1.4	3.4 ± 1.3
Time since diagnosis, years	1.66 ± 1.3	1.4 ± 1.1
PSPRS total score	36.7 ± 10.3	36.4 ± 11.5

*Note*: Values are expressed as mean ± standard deviation unless otherwise specified.

Abbreviation: PSPRS, progressive supranuclear palsy rating scale.

**TABLE 2:** Cognitive subtest completion in PASSPORT clinical trial.

Cognitive test	Baseline (*n* = 486)	Week 12 (*n* = 471)	Week 24 (*n* = 460)	Week 36 (*n* = 439)	Week 48 (*n* = 422)	Week 52 (*n* = 403)
RBANS‐coding	465	430	410	381	–	353
RBANS‐digit span	484	459	443	421	–	395
RBANS‐figure copy	476	457	438	409	–	390
RBANS‐figure recall	476	456	434	403	–	384
RBANS‐line orientation	481	456	438	415	–	388
RBANS‐list learning	483	460	443	423	–	403
RBANS‐list recall	484	459	441	420	–	397
RBANS‐list recognition	483	458	439	418	–	395
RBANS‐picture naming	485	460	444	423	–	398
RBANS‐semantic fluency	484	459	444	421	–	399
RBANS‐story memory	484	459	443	422	–	402
RBANS‐story recall	484	459	441	418	–	395
Color trails 1	372	327	295	274	248	–
Color trails 2	268	245	212	194	180	–
Letter‐number sequencing	473	451	433	417	409	–
Phonemic fluency	486	459	445	423	414	–

Abbreviation: RBANS, Repeatable Battery for the Assessment of Neuropsychological Status.

**FIGURE 1:**
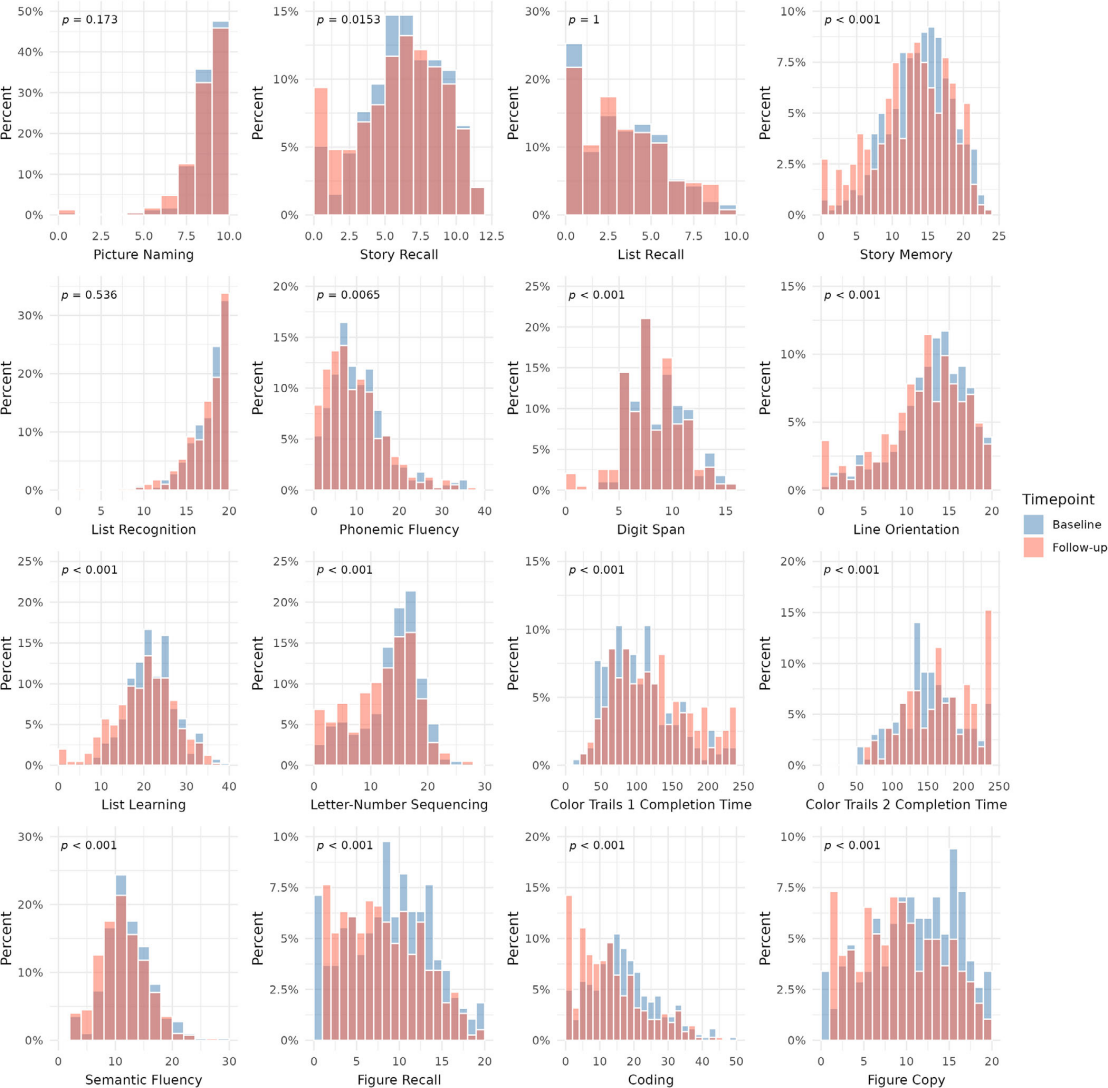
Histograms of raw cognitive test scores at baseline and final follow‐up (week 48 or 52) in the PASSPORT clinical trial. Lower scores indicate worse performance for all measures except for Color Trails 1 and 2, for which higher scores reflect worsening (i.e., longer completion time). Only participants with measurements at both time points are included. Paired *t*‐tests assess a significant difference between scores at each time point, and Bonferroni‐adjusted *p* values are reported.

Paired *t*‐tests reveal significant differences between cognitive test scores at baseline and final follow‐up (week 52 or 48) for all cognitive tests except picture naming, list recall, and list recognition after Bonferroni correction (Figure 1, Table 3). The largest magnitude effect sizes were observed for figure copy (Cohen's *d* = −0.658) and coding (Cohen's *d* = −0.616), and the smallest magnitude effect sizes were observed for list recall (Cohen's *d* = 0.072) and list recognition (Cohen's *d* = 0.110) (Table 3). As visualized in the histograms, score distributions for picture naming and list recognition exhibited a ceiling effect at both time points, while score distributions for list recall, coding, phonemic fluency, and Color Trails 2 exhibited a floor effect at the final time point (Figure 1). While there existed inter‐patient variability, change in subtest score between baseline and final follow‐up was comparable (Figure S1). The paired *t*‐test assumption of normality in differences between paired values was met for all subtests. Wilcoxon signed‐rank tests yielded comparable results.

**TABLE 3 alz71641-tbl-0003:** Paired *t*‐tests comparing cognitive subtest scores at baseline and final follow‐up.

Cognitive test	*N*	Baseline mean	Final follow‐up mean	Mean difference	Cohen's *d*	*p* value
RBANS‐coding	344	17.36 ± 9.55	13.47 ± 10.13	−3.89 ± 6.32	−0.616	<0.001
RBANS‐digit span	394	9.15 ± 2.50	8.64 ± 2.77	−0.51 ± 2.24	−0.227	<0.001
RBANS‐figure copy	383	11.30 ± 5.12	8.48 ± 6.63	−2.82 ± 4.29	−0.658	<0.001
RBANS‐figure recall	379	9.24 ± 4.94	7.29 ± 5.12	−1.95 ± 4.10	−0.476	<0.001
RBANS‐line orientation	384	13.50 ± 4.13	12.60 ± 4.77	−0.90 ± 3.97	−0.227	<0.001
RBANS‐list learning	402	22.25 ± 5.67	20.64 ± 7.11	−1.61 ± 4.70	−0.342	<0.001
RBANS‐list recall	396	3.62 ± 2.60	3.77 ± 2.59	0.15 ± 2.22	0.072	1.00
RBANS‐list recognition	393	18.17 ± 2.02	17.95 ± 2.40	−0.22 ± 1.97	−0.110	0.536
RBANS‐picture naming	397	9.19 ± 1.16	9.01 ± 1.51	−0.18 ± 1.41	−0.131	0.173
RBANS‐semantic fluency	398	12.69 ± 4.02	11.79 ± 4.04	−0.89 ± 3.25	−0.275	<0.001
RBANS‐story memory	401	14.33 ± 4.47	13.24 ± 5.33	−1.08 ± 4.03	−0.269	<0.001
RBANS‐story recall	394	6.87 ± 2.75	6.44 ± 3.15	−0.43 ± 2.56	−0.169	0.015
Color Trails 1	233	105.05 ± 47.45	123.10 ± 53.99	18.06 ± 37.28	0.484	<0.001
Color Trails 2	164	153.31 ± 44.07	168.67 ± 47.78	15.36 ± 36.81	0.417	<0.001
Letter‐Number Sequencing	393	5.26 ± 12.52	12.52 ± 5.86	−1.67 ± 4.32	−0.387	<0.001
Phonemic fluency	395	10.16 ± 6.78	10.16 ± 6.78	−0.90 ± 4.99	−0.181	0.007

*Note*: Paired *t*‐tests were conducted for each cognitive subtest to compare scores at baseline and final follow‐up. Only participants with scores at both time points were included. Bonferroni‐adjusted *p* values are reported. Values are expressed as mean ± standard deviation unless otherwise specified. Final follow‐up occurred at week 52 for RBANS subtests and at week 48 for Color Trails tests, Letter‐Number Sequencing, and phonemic fluency. For RBANS subtests, Letter‐Number Sequencing, and phonemic fluency, negative Cohen's *d* values indicate worsening from baseline, whereas for Color Trails tests, positive Cohen's *d* values indicate worsening.

Abbreviation: RBANS, Repeatable Battery for the Assessment of Neuropsychological Status.

**FIGURE 2 alz71641-fig-0002:**
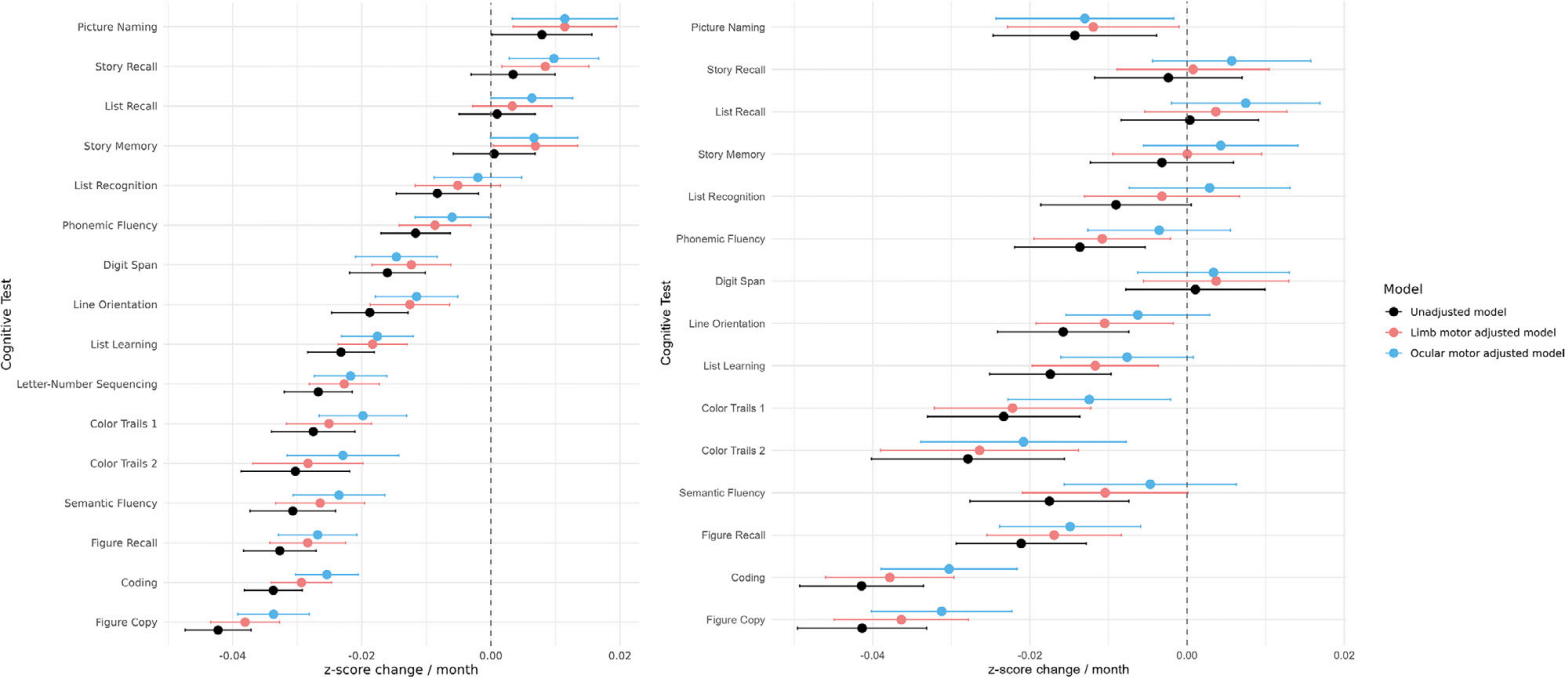
(A and B) Forest plots depicting monthly change in *z*‐score in each cognitive outcome measure, estimated using LMMs controlling for years since diagnosis, baseline age group, sex, and treatment arm. Plots for the PASSPORT (left) and tilavonemab (right) clinical trial datasets are shown, ordered by decreasing monthly *z*‐score change in the PASSPORT dataset. Letter‐Number Sequencing was not included in the tilavonemab clinical trial. The limb motor and ocular motor adjusted models include limb motor and ocular motor subscores from the PSP Rating Scale as additional covariates, respectively. All participant data, regardless of study completion status, are included.

LMM analyses of the PASSPORT dataset indicate that the largest magnitude *z*‐score changes per month occurred in figure copy (−0.042, *p* < 0.001), coding, (−0.034, *p* < 0.001), figure recall (−0.033, *p* < 0.001), semantic fluency (−0.031, *p* < 0.001), Color Trails 2 (−0.030, *p* < 0.001), and Color Trails 1 (−0.028, *p* < 0.001) (Figure 2A, Table 4). After Bonferroni correction, there was no significant *z*‐score change per month for list recall (0.001, *p* = 1.0), list recognition (−0.008, *p* = 0.171), story memory (0.001, *p* = 1.0), story recall (0.003, *p* = 1.0), or picture naming (0.008, *p* = 0.731) (Figure 2A, Table 4). Sensitivity analyses restricted to participants who completed the double‐blind period yielded similar results, supporting inclusion of all participant data in this analysis (Figure S2).

**TABLE 4 alz71641-tbl-0004:** Monthly change in cognitive subtest scores in PASSPORT and tilavonemab (ABBV‐8E12) clinical trials.

	PASSPORT clinical trail			Tilavonemab (ABBV‐8E12) clinical trial		
	Estimate	Standard error	*p* value	Estimate	Standard error	*p* value
RBANS‐coding	−0.034	0.0023	<0.001	−0.041	0.0040	<0.001
RBANS‐digit span	−0.016	0.0030	<0.001	0.001	0.0045	1.00
RBANS‐figure copy	−0.042	0.0026	<0.001	−0.041	0.0042	<0.001
RBANS‐figure recall	−0.033	0.0029	<0.001	−0.021	0.0042	<0.001
RBANS‐line orientation	−0.019	0.0030	<0.001	−0.016	0.0043	0.003
RBANS‐list learning	−0.024	0.0032	<0.001	−0.017	0.0039	<0.001
RBANS‐list recall	0.001	0.0030	1.00	0.0004	0.0045	1.00
RBANS‐list recognition	−0.008	0.0033	0.171	−0.009	0.0049	1.00
RBANS‐picture naming	0.008	0.0040	0.731	−0.014	0.0053	0.109
RBANS‐semantic fluency	−0.031	0.0034	<0.001	−0.018	0.0052	0.011
RBANS‐story memory	0.001	0.0032	1.00	−0.003	0.0046	1.00
RBANS‐story recall	0.003	0.0033	1.00	−0.002	0.0048	1.00
Color Trails 1	−0.028	0.0033	<0.001	−0.023	0.0049	<0.001
Color Trails 2	−0.030	0.0043	<0.001	−0.028	0.0063	<0.001
Letter‐Number Sequencing	−0.027	0.0027	<0.001	N/A	N/A	N/A
Phonemic fluency	−0.012	0.0027	<0.001	−0.014	0.0043	0.020

*Note*: Estimates, standard errors, and Bonferroni‐adjusted *p* values were derived from linear mixed‐effects models of cognitive subtest scores, adjusted for years since diagnosis, baseline age group, sex, and treatment arm. Letter‐Number Sequencing was not included in the tilavonemab (ABBV‐8E12) dataset.

Abbreviation: RBANS, Repeatable Battery for the Assessment of Neuropsychological Status.

Results are highly consistent between the PASSPORT and tilavonemab datasets. The same six cognitive tests exhibited the largest magnitude monthly *z*‐score declines in both datasets, although their relative order differs (Figure 2, Table 4). In the tilavonemab dataset, coding declined more rapidly (−0.041, *p* < 0.001), whereas semantic fluency (−0.018, *p* = 0.011) and figure recall (−0.021, *p* < 0.001) exhibited more modest declines. List recall, list recognition, story memory, story recall, and picture naming showed no significant change in either dataset. In the tilavonemab dataset, digit span also exhibited non‐significant *z*‐score change per month (Figure 2B, Table 4). Overall, the results were comparable across the two datasets, although effect sizes were generally smaller in the tilavonemab dataset and standard errors were larger, a likely consequence of the trial's smaller sample size.

The sensitivity analyses controlling for limb motor or ocular motor disability showed reductions in the magnitude of monthly *z*‐score decline across most cognitive tests and in both datasets, indicating that motor impairment partially explains the observed decline in cognitive test scores (Figure 2, Tables S1 and S2). In the PASSPORT dataset, after adjusting for ocular motor impairment, monthly change in phonemic fluency *z*‐score was no longer significant (Table S1). In the tilavonemab dataset, adjusting for either limb motor or ocular motor impairment resulted in non‐significant monthly change in line orientation, list learning, semantic fluency, and phonemic fluency; adjusting for ocular motor impairment also rendered monthly change in Color Trails 1 non‐significant (Table S2). However, these findings may reflect smaller sample sizes and larger standard errors in the tilavonemab dataset relative to PASSPORT. Exploratory analyses of PSPRS item‐level covariates in the PASSPORT dataset suggested that impairment of downward saccades, left and right saccades, and finger tapping contributed to large reductions in the magnitude of monthly *z*‐score (with considerable variability across subtests), whereas eyelid dysfunction, limb rigidity, and tremor had minimal impacts on monthly change (Table S3).

## 4 DISCUSSION

This study adds to the relatively limited body of literature on cognition in PSP by examining longitudinal performance on 16 cognitive outcome measures in two large, well‐characterized clinical trial cohorts. We found that subtests primarily assessing memory (particularly list recall, list recognition, story memory, and story recall) exhibited minimal and non‐significant change over time. This finding is consistent across both immediate and delayed memory tests and aligns with prior research on memory in PSP,^5^, ^6^ indicating that memory declines relatively slowly as the disease progresses. Interestingly, not all memory‐assessing outcome measures displayed the same behavior. We observed a ceiling effect for list recognition at both time points, showing that recognition capabilities are relatively intact in PSP. In contrast, a floor effect existed for list recall at both time points, making it difficult to detect a longitudinal change in this subtest. Together, these findings suggest that PSP recall deficits are more likely driven by impairment in retrieval compared to deficits in recognition.

Coding and both Color Trails Tests significantly declined, consistent with the previously reported decline in Color Trails 2 in the davunetide clinical trial.^20^ However, Color Trails 2 was subject to a floor effect and a high non‐completion percentage. All three tests assess complex cognitive functions including working memory, visuospatial skills, visual processing, processing speed, selective and divided attention, psychomotor coordination, and executive function.^16^, ^30^ Our findings suggest that the RBANS coding subtest may be an alternative to Color Trails in future clinical trials due to its higher completion rates and larger observed rate of decline, indicating that it is highly sensitive to PSP cognitive changes. Nevertheless, coding is similarly subject to a floor effect after 1 year and demonstrates confounding by ocular motor and motor dysfunction. Unlike Color Trails, however, the coding test can be administered orally, which may reduce confounding due to motor disability.

We found that fluency was also significantly impaired in PSP, in agreement with prior literature.^5^, ^9^, ^31^, ^32^ Both semantic (categorical) and phonemic (letter) fluency declined significantly over time. Prior research emphasized phonemic fluency deficiencies in PSP over those of semantic fluency.^9^, ^31^, ^32^ We observed greater impairment of phonemic fluency at baseline, while semantic fluency declined more rapidly over 1 year. This likely reflects a floor effect in phonemic fluency after 1 year, limiting its ability to detect longitudinal change. These findings indicate that measures of semantic fluency may better track cognitive decline than measures of phonemic fluency because of greater phonemic fluency impairment at earlier disease stages. We should note, however, that semantic fluency shows inconsistency across the two datasets: Its rate of monthly decline was smaller in the tilavonemab cohort and became non‐significant after adjustment for motor disability. This highlights the need to validate the trajectory of semantic fluency decline in additional PSP cohorts. The other subtest in the RBANS language domain, picture naming, did not demonstrate significant change over a 1‐year period.

Consistent[Table alz71641-tbl-0003] with[Fig alz71641-fig-0002], [Table alz71641-tbl-0004] prior research showing a decline in visuospatial function in PSP,^11^, ^33^ figure copy and figure recall exhibited two of the fastest rates of change in both datasets, suggesting a large reduction in visuospatial skills as the disease progresses. However, these measures may be highly susceptible to confounding by motor and ocular motor impairment. Studies of an alternative measure of visuospatial function, the visual object and space perception (VOSP) battery, similarly noted confounding by ocular motor impairment.^33^ In our sensitivity analysis, adjusting for limb motor and ocular motor impairment attenuated the rate of decline across most cognitive measures. In agreement with clinical expectations, the effect of ocular motor impairment was highly pronounced for subtests requiring visual scanning and attention, including figure copy, coding, and Color Trails. Our exploratory analysis suggests that impairment of eye movements (e.g., vertical and horizontal saccades) and fine motor skills (e.g., finger tapping) may interfere with accurate measurement of cognitive decline in PSP.

This study is subject to several limitations. First, participants were enrolled at a relatively early stage of the disease, with a mean baseline PSPRS score of 36.7 and 36.4 in the PASSPORT and tilavonemab trials, respectively.^26^, ^27^ We would not expect more advanced PSP patients to perform similarly in cognitive testing, nor is it known whether cognitive decline is linear as the disease progresses. Second, both trials limited eligibility to patients with symptom duration under 5 years. This criterion eliminates participants with longer disease duration and potentially slower rates of disease progression. Third, both clinical trials primarily included patients with PSP Richardson syndrome, limiting the generalizability of our findings to other PSP phenotypes. Fourth, participants in both cohorts were highly educated and White (86.2% and 85.9% White non‐Hispanic in the PASSPORT and tilavonemab trials, respectively),^26^, ^27^ and thus the clinical trial populations may not be representative of the larger PSP population. Fifth, we would have preferred to control for patient symptom duration in our analysis, which may be a better measure of disease duration than years since diagnosis. This information, although collected in the trials, was not available to us. Sixth, participant education level was unfortunately not available. Seventh, we are aware that adjusting for limb motor and ocular motor scores does not adequately address the question of confounding by motor disability. Since these measures progress in parallel, we may be detecting collinearity. However, we encourage future trials to try to reduce the impact of motor disability on cognitive measurement, for example by administering scales orally when possible.

Regardless of these limitations, this study provides a novel longitudinal analysis of cognitive measures in two large well‐characterized cohorts of patients with PSP. Our findings may help improve clinical trial design through the incorporation of cognitive outcome measures that are more sensitive and specific to the cognitive changes in PSP. Future analyses will be needed to expand to more generalized PSP populations.

## AUTHOR CONTRIBUTIONS

**Zoë C. Cooper and Anne‐Marie Wills**: conceptualization. **Zoë C. Cooper, Douglas Gunzler, and Anne‐Marie Wills**: methodology. **Zoë Cooper and Indira Garcia‐Cordero**: data curation and formal analysis. **Zoë Cooper**: writing – original draft. **Indira Garcia‐Cordero, Maria Carmela Tartaglia, Adam M. Staffaroni, Kevin Duff, Douglas Gunzler, Adam L. Boxer, and Anne‐Marie Wills**: writing – review and editing.

## CONFLICT OF INTEREST STATEMENT

I.G.C. is funded by the AFTD Pathways for Hope Pilot Grant (2025‐002). M.C.T. is a member of the Scientific Advisory Board for the Women's Brain Project, PSP Canada, and Brain Injury Canada. She has participated in clinical trials funded by Avanex, Biogen, BMS, Green Valley, GSK, Janssen, Merck, Novo Nordisk, Passage Bio, and UCB and has served as a consultant for Eisai, Eli Lilly, and Novo Nordisk. She is supported by the Marion and Gerald Soloway Chair in Brain Injury and Concussion Research. A.M.S. has received research funding from National Institute on Aging (NIA)/National Institutes of Health (NIH) Awards RF1AG077557 and K23AG061253, the Association for Frontemporal Degeneration, the ALS Association, Bluefield Project to Cure FTD, the Alzheimer's Association, and the Rainwater Charitable Foundation. He has provided consulting for Alector Inc., Aviado Bio, CervoMed, Coya, Eli Lilly, and Takeda. He is a co‐inventor of digital cognitive tasks that have been licensed through UCSF. A.L.B. has served as a paid consultant to Alector, Alexion, Arrowhead, Arvinas, BMS, Eli Lilly, Janssen, Merck, Neurocrine, Novartis, Oligomerix, Ono, Oscotec, Switch, and Transposon. He is a scientific cofounder of Neurovanda. His institution received research support from Biogen, Eisai, and Regeneron for serving as a site investigator for clinical trials. A.M.W. has research funding from NIH/NIH Award R44AG080861 and NIA/NIH Award R01AG085029, from the Parkinson's Foundation, and has participated in clinical trials funded by Roche/Genentech, Biogen/Denali, Bial, Amylyx, Ferrer, Ono, and Biohaven. She has received consultant payments from Accordant, CVS/Caremark, Genentech, Amylyx, and Ono. All remaining authors declare no competing interests. Author disclosures are available in the supporting information. Author disclosures are available in the Supporting Information.

## CONSENT STATEMENT

Participants and their study partners provided written informed consent prior to screening and all study procedures. The study obtained approval from an independent ethics committee or Institutional Review Board at each participating site and was conducted in accordance with the ethical standards of the Declaration of Helsinki.

## Supporting information




Supporting Information

Supporting Information

## Data Availability

Publicly available datasets were analyzed in this study. Data can be found at https://portal.rdca.c‐path.org/ and https://vivli.org. Data were under license for this study.
