# Supplementary material for: Longitudinal cognitive outcomes in two progressive supranuclear palsy clinical trials
Source: Alzheimers Dement. 2026 Jun 26;22(7):e71641. doi: 10.1002/alz.71641 (PMC13309287; doi:10.1002/alz.71641)
Supplement: Supplementary file 2 — Supporting Information [file ALZ-22-e71641-s001.docx]

Supplementary Table 1. Monthly change in cognitive subtest scores after adjustment for ocular motor and limb motor disability in the PASSPORT clinical trial

|  | Unadjusted model | | | Ocular motor adjusted model | | | Limb motor adjusted model | | |
| --- | --- | --- | --- | --- | --- | --- | --- | --- | --- |
|  | Estimate | Standard error | *P*-value | Estimate | Standard error | *P*-value | Estimate | Standard error | *P*-value |
| RBANS-Coding | -0.034 | 0.0023 | <0.001 | -0.025 | 0.0025 | <0.001 | -0.029 | 0.0024 | <0.001 |
| RBANS-Digit Span | -0.016 | 0.0030 | <0.001 | -0.015 | 0.0032 | <0.001 | -0.012 | 0.0031 | 0.001 |
| RBANS-Figure Copy | -0.042 | 0.0026 | <0.001 | -0.034 | 0.0028 | <0.001 | -0.038 | 0.0027 | <0.001 |
| RBANS-Figure Recall | -0.033 | 0.0029 | <0.001 | -0.027 | 0.0031 | <0.001 | -0.028 | 0.0030 | <0.001 |
| RBANS-Line Orientation | -0.019 | 0.0030 | <0.001 | -0.012 | 0.0028 | 0.00663 | -0.013 | 0.0031 | 0.001 |
| RBANS-List Learning | -0.024 | 0.0032 | <0.001 | -0.018 | 0.0029 | <0.001 | -0.018 | 0.0027 | <0.001 |
| RBANS-List Recall | 0.001 | 0.0030 | 1.00 | 0.006 | 0.0032 | 0.793 | 0.003 | 0.0031 | 1.0 |
| RBANS-List Recognition | -0.008 | 0.0033 | 0.171 | -0.003 | 0.0035 | 1.0 | -0.005 | 0.0034 | 1.0 |
| RBANS-Picture Naming | 0.008 | 0.0040 | 0.731 | 0.011 | 0.0042 | 0.0959 | 0.011 | 0.0041 | 0.082 |
| RBANS-Semantic Fluency | -0.031 | 0.0034 | <0.001 | -0.024 | 0.0036 | <0.001 | -0.026 | 0.0035 | <0.001 |
| RBANS-Story Memory | 0.001 | 0.0032 | 1.00 | 0.007 | 0.0035 | 0.854 | 0.007 | 0.0033 | 0.631 |
| RBANS-Story Recall | 0.003 | 0.0033 | 1.00 | 0.010 | 0.0035 | 0.0932 | 0.008 | 0.0034 | 0.227 |
| Color Trails 1 | -0.028 | 0.0033 | <0.001 | -0.020 | 0.0035 | <0.001 | -0.025 | 0.0034 | <0.001 |
| Color Trails 2 | -0.030 | 0.0043 | <0.001 | -0.023 | 0.0044 | <0.001 | -0.028 | 0.0044 | <0.001 |
| Letter-Number Sequencing | -0.027 | 0.0027 | <0.001 | -0.022 | 0.0029 | <0.001 | -0.023 | 0.0028 | <0.001 |
| Phonemic Fluency | -0.012 | 0.0027 | <0.001 | -0.006 | 0.0030 | 0.643 | -0.009 | 0.0028 | 0.036 |

Estimates, standard errors, and Bonferroni-adjusted p-values were derived from linear mixed-effects models of cognitive subtest scores, adjusted for years since diagnosis, baseline age group, sex, and treatment arm. The ocular motor–adjusted model additionally included the ocular motor domain of the PSP Rating Scale as a covariate, and the limb motor–adjusted model additionally included the limb motor domain.

Abbreviations: RBANS, Repeatable Battery for the Assessment of Neuropsychological Status

Supplementary Table 2. Monthly change in cognitive subtest scores after adjustment for ocular motor and limb motor disability in the Tilavonemab (ABBV-8E12) clinical trial

|  | Unadjusted model | | | Ocular motor adjusted model | | | Limb motor adjusted model | | |
| --- | --- | --- | --- | --- | --- | --- | --- | --- | --- |
|  | Estimate | Standard Error | *P*-Value | Estimate | Standard Error | *P*-Value | Estimate | Standard Error | *P*-Value |
| RBANS-Coding | -0.041 | 0.0040 | <0.001 | -0.030 | 0.0044 | <0.001 | -0.038 | 0.0046 | <0.001 |
| RBANS-Digit Span | 0.001 | 0.0045 | 1.00 | 0.003 | 0.0049 | 1.00 | 0.004 | 0.0047 | 1.00 |
| RBANS-Figure Copy | -0.041 | 0.0042 | <0.001 | -0.0312 | 0.0046 | <0.001 | -0.036 | 0.0044 | <0.001 |
| RBANS-Figure Recall | -0.021 | 0.0042 | <0.001 | -0.015 | 0.0046 | 0.018 | -0.017 | 0.0044 | 0.002 |
| RBANS-Line Orientation | -0.016 | 0.0043 | 0.003 | -0.0063 | 0.0047 | 1.00 | -0.010 | 0.0044 | 0.279 |
| RBANS-List Learning | -0.017 | 0.0039 | <0.001 | -0.008 | 0.0043 | 1.00 | -0.0120 | 0.0041 | 0.067 |
| RBANS-List Recall | 0.000 | 0.0045 | 1.00 | 0.007 | 0.0048 | 1.00 | 0.004 | 0.0046 | 1.00 |
| RBANS-List Recognition | -0.009 | 0.0049 | 1.00 | 0.003 | 0.0052 | 1.00 | -0.003 | 0.0050 | 1.00 |
| RBANS-Picture Naming | -0.014 | 0.0053 | 0.109 | -0.013 | 0.0058 | 0.366 | -0.012 | 0.0056 | 0.486 |
| RBANS-Semantic Fluency | -0.018 | 0.0052 | 0.011 | -0.005 | 0.0056 | 1.00 | -0.010 | 0.0054 | 0.794 |
| RBANS-Story Memory | -0.003 | 0.0046 | 1.00 | 0.004 | 0.0050 | 1.00 | 0.000 | 0.0048 | 1.00 |
| RBANS-Story Recall | -0.002 | 0.0048 | 1.00 | 0.006 | 0.0051 | 1.00 | 0.001 | 0.0049 | 1.00 |
| Color Trails 1 | -0.023 | 0.0049 | <0.001 | -0.012 | 0.0053 | 0.279 | -0.022 | 0.0051 | <0.001 |
| Color Trails 2 | -0.028 | 0.0063 | <0.001 | -0.021 | 0.0067 | 0.028 | -0.026 | 0.0064 | <0.001 |
| Phonemic Fluency | -0.014 | 0.0043 | 0.020 | -0.004 | 0.0046 | 1.00 | -0.011 | 0.0044 | 0.226 |

Estimates, standard errors, and Bonferroni-adjusted p-values were derived from linear mixed-effects models of cognitive subtest scores, adjusted for years since diagnosis, baseline age group, sex, and treatment arm. The ocular motor–adjusted model additionally included the ocular motor domain of the PSP Rating Scale as a covariate, and the limb motor–adjusted model additionally included the limb motor domain.

Abbreviations: RBANS, Repeatable Battery for the Assessment of Neuropsychological Status

Supplementary Table 3. Monthly change in cognitive subtest scores after adjustment by individual PSP Rating Scale ocular motor and limb motor items

|  | Main Model | PSPRS Ocular Motor Domain | | | | PSPRS Limb Motor Domain | | | | | |
| --- | --- | --- | --- | --- | --- | --- | --- | --- | --- | --- | --- |
|  |  | Upward Saccades | Downward Scaccades | Left & Right Saccades | Eyelid Dysfunction | Limb Rigidity | Limb Dystonia | Finger Tapping | Toe Tapping | Apraxia of Hand Movement | Tremor |
| RBANS-Coding | -0.034 | -0.031 | -0.029 | -0.030 | -0.032 | -0.032 | -0.032 | -0.032 | -0.032 | -0.033 | -0.034 |
| RBANS-Digit Span | -0.016 | -0.015 | -0.015 | -0.015 | -0.016 | -0.015 | -0.015 | -0.014 | -0.015 | -0.014 | -0.016 |
| RBANS-Figure Copy | -0.042 | -0.039 | -0.037 | -0.038 | -0.040 | -0.041 | -0.040 | -0.041 | -0.041 | -0.041 | -0.042 |
| RBANS-Figure Recall | -0.033 | -0.031 | -0.029 | -0.029 | -0.032 | -0.032 | -0.030 | -0.031 | -0.031 | -0.032 | -0.033 |
| RBANS-Line Orientation | -0.019 | -0.017 | -0.015 | -0.014 | -0.016 | -0.017 | -0.017 | -0.017 | -0.017 | -0.016 | -0.019 |
| RBANS-List Learning | -0.024 | -0.021 | -0.019 | -0.020 | -0.022 | -0.021 | -0.022 | -0.021 | -0.022 | -0.021 | -0.023 |
| RBANS-List Recall | 0.001 | 0.003 | 0.004 | 0.005 | 0.003 | 0.002 | 0.001 | 0.003 | 0.002 | 0.002 | 0.001 |
| RBANS-List Recognition | -0.008 | -0.005 | -0.004 | -0.005 | -0.007 | -0.007 | -0.008 | -0.007 | -0.007 | -0.006 | -0.008 |
| RBANS-Picture Naming | 0.008 | 0.010 | 0.011 | 0.011 | 0.007 | 0.009 | 0.010 | 0.009 | 0.009 | 0.010 | 0.008 |
| RBANS-Semantic Fluency | -0.031 | -0.027 | -0.026 | -0.027 | -0.030 | -0.029 | -0.030 | -0.029 | -0.029 | -0.029 | -0.031 |
| RBANS-Story Memory | 0.001 | 0.003 | 0.004 | 0.005 | 0.002 | 0.003 | 0.002 | 0.004 | 0.002 | 0.004 | 0.001 |
| RBANS-Story Recall | 0.003 | 0.006 | 0.008 | 0.007 | 0.004 | 0.005 | 0.006 | 0.006 | 0.005 | .006 | 0.004 |
| Color Trails 1 | -0.028 | -0.024 | -0.021 | -0.025 | -0.028 | -0.027 | -0.027 | -0.027 | -0.027 | -0.027 | -0.028 |
| Color Trails 2 | -0.030 | -0.027 | -0.025 | -0.028 | -0.028 | -0.029 | -0.030 | -0.029 | -0.030 | -0.029 | -0.030 |
| Letter-Number Sequencing | -0.027 | -0.026 | -0.023 | -0.023 | -0.027 | 0.026 | -0.026 | -0.025 | -0.025 | -0.025 | -0.027 |
| Phonemic Fluency | -0.012 | -0.009 | -0.007 | -0.009 | -0.012 | -0.011 | -0.010 | -0.010 | -0.011 | -0.012 | -0.012 |

| Percent Change Key | 0-5% | 5-10% | 10-15% | 15-20% | 20-25% |
| --- | --- | --- | --- | --- | --- |

Estimates were derived from linear mixed-effects models of cognitive subtest scores, adjusted for years since diagnosis, baseline age group, sex, treatment arm, and a treatment arm by time interaction. Models were fitted to adjust for individual items of the PSP rating scale ocular motor and limb motor domains. Percent change is visualized relative to the main model, only for subtests with absolute value of the main model estimate ≥ 0.02, due to stability of percent change calculation.

Abbreviations: PSP, progressive supranuclear palsy; RBANS, Repeatable Battery for the Assessment of Neuropsychological Status


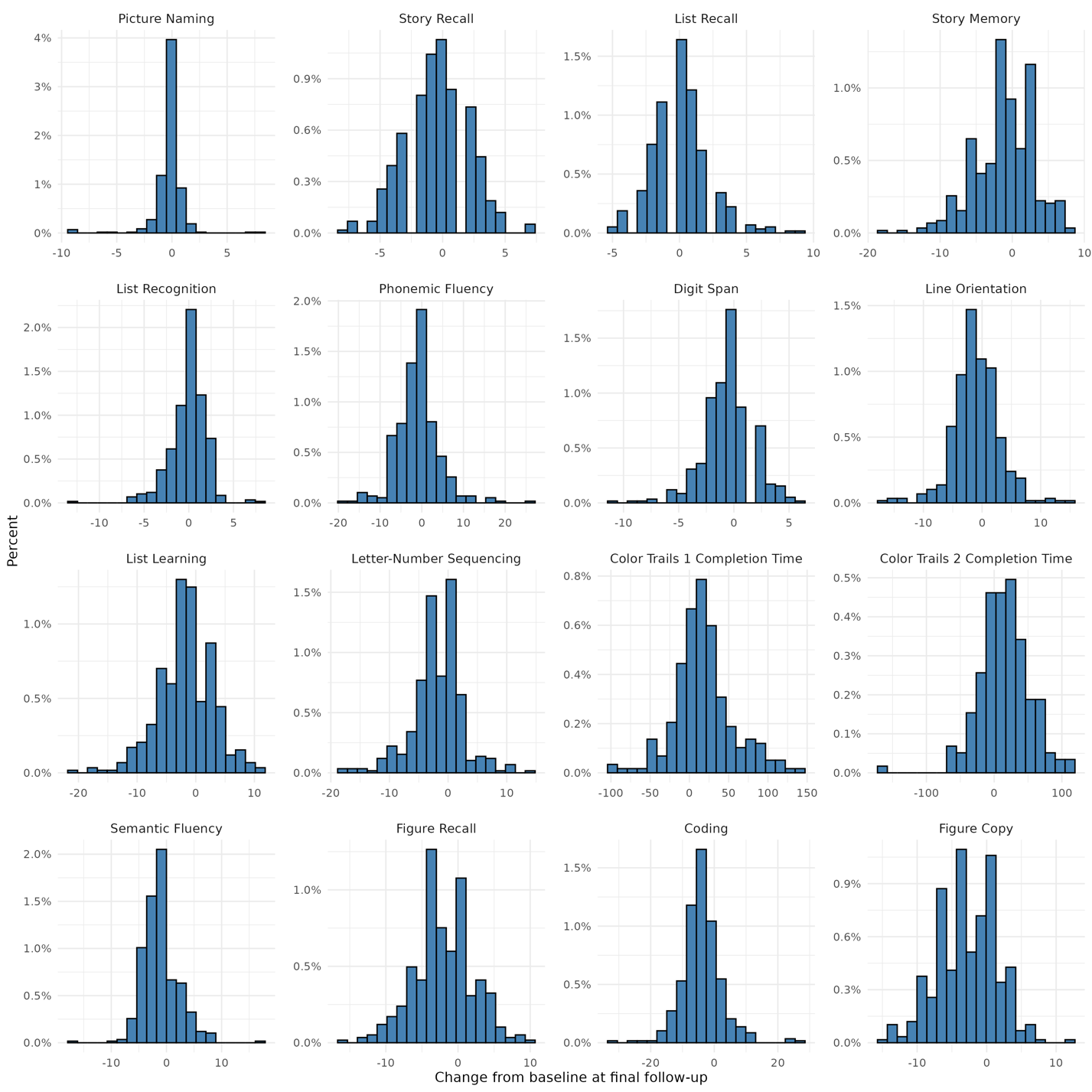


Supplementary FIGURE 1

Histograms of cognitive test score change between baseline and final follow-up (week 48 or 52) in the PASSPORT clinical trial. Negative change indicates worsening performance for all measures except for Color Trails 1 and 2, for which positive change reflects worsening (i.e. longer completion time). Only participants with measurements at both timepoints are included.


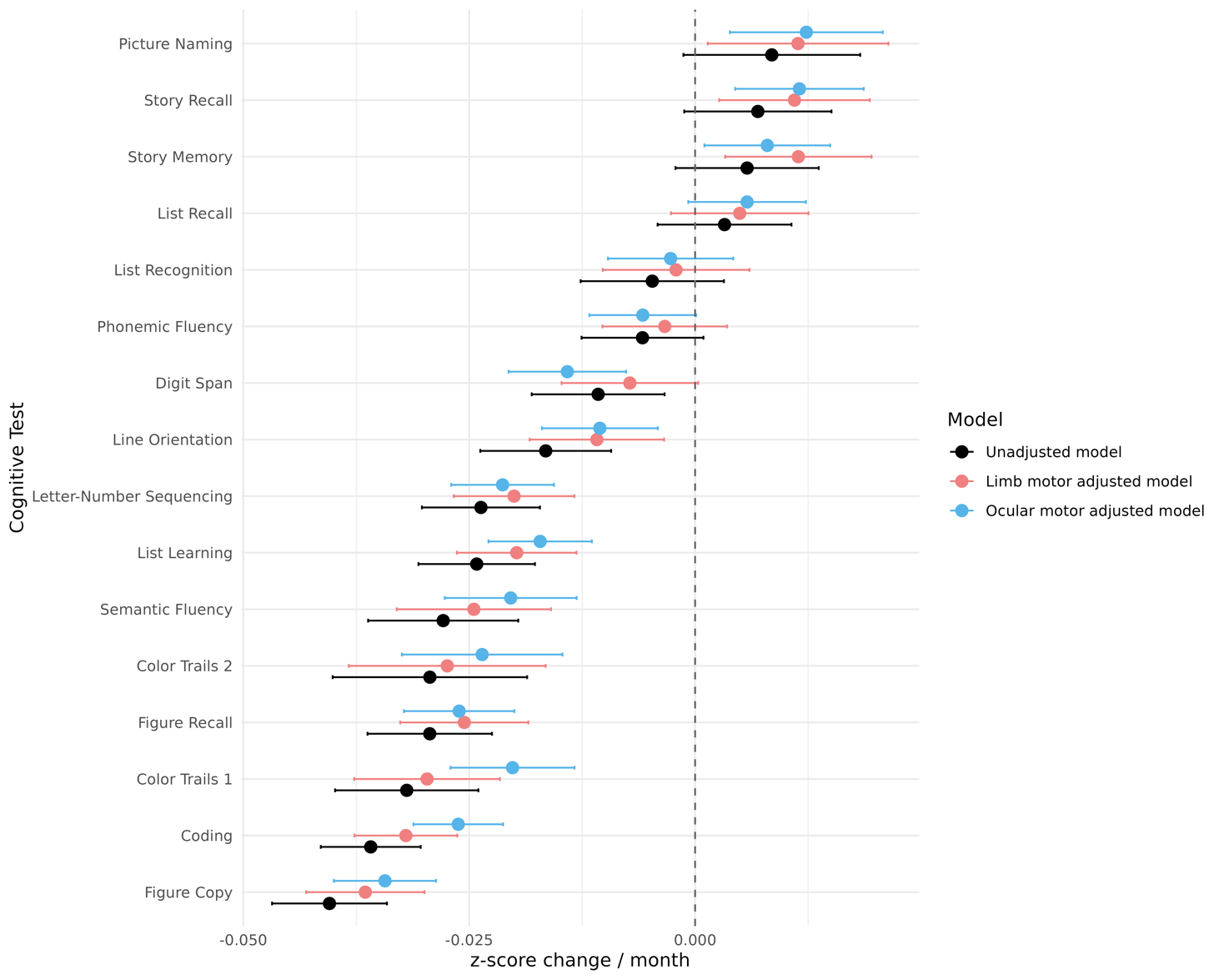


Supplementary FIGURE 2

Forest plot of monthly change in z-score in each cognitive outcome measure, restricted to participants who completed the double-blind period of the PASSPORT trial. Estimates are derived from on linear mixed-effects models controlling for years since diagnosis, baseline age group, sex, and treatment arm. The limb motor and ocular motor adjusted models include limb motor and ocular motor sub-scores from the PSP Rating Scale as additional covariates, respectively.
